# Metabolite profile of a mouse model of Charcot–Marie–Tooth type 2D neuropathy: implications for disease mechanisms and interventions

**DOI:** 10.1242/bio.019273

**Published:** 2016-06-10

**Authors:** Preeti Bais, Kirk Beebe, Kathryn H. Morelli, Meagan E. Currie, Sara N. Norberg, Alexei V. Evsikov, Kathy E. Miers, Kevin L. Seburn, Velina Guergueltcheva, Ivo Kremensky, Albena Jordanova, Carol J. Bult, Robert W. Burgess

**Affiliations:** 1 The Jackson Laboratory, Bar Harbor, 04609 ME, USA; 2 Metabolon Inc., Durham, 27713 NC, USA; 3 Graduate School of Biomedical Science and Engineering, University of Maine, Orono, 04469 ME, USA; 4 Department of Molecular Medicine, USF Health, University of South Florida, Tampa, 33620 FL, USA; 5 Department of Neurology, Medical University-Sofia, 1431 Sofia, Bulgaria; 6 National Genetics Laboratory, Department of Obstetrics and Gynecology, University Hospital of Obstetrics and Gynecology, Medical University-Sofia, 1431 Sofia, Bulgaria; 7 Molecular Neurogenomics Group, VIB Department of Molecular Genetics, University of Antwerp, 2610 Antwerpen, Belgium; 8 Molecular Medicine Center, Department of Medical Chemistry and Biochemistry, Medical University-Sofia, 1431 Sofia, Bulgaria

**Keywords:** Peripheral neuropathy, Spinal cord, Sciatic nerve, Metabolomics, Mass Spectrometry, tRNA synthetase

## Abstract

Charcot–Marie–Tooth disease encompasses a genetically heterogeneous class of heritable polyneuropathies that result in axonal degeneration in the peripheral nervous system. Charcot–Marie–Tooth type 2D neuropathy (CMT2D) is caused by dominant mutations in glycyl tRNA synthetase (*GARS*). Mutations in the mouse *Gars* gene result in a genetically and phenotypically valid animal model of CMT2D. How mutations in *GARS* lead to peripheral neuropathy remains controversial. To identify putative disease mechanisms, we compared metabolites isolated from the spinal cord of *Gars* mutant mice and their littermate controls. A profile of altered metabolites that distinguish the affected and unaffected tissue was determined. Ascorbic acid was decreased fourfold in the spinal cord of CMT2D mice, but was not altered in serum. Carnitine and its derivatives were also significantly reduced in spinal cord tissue of mutant mice, whereas glycine was elevated. Dietary supplementation with acetyl-L-carnitine improved gross motor performance of CMT2D mice, but neither acetyl-L-carnitine nor glycine supplementation altered the parameters directly assessing neuropathy. Other metabolite changes suggestive of liver and kidney dysfunction in the CMT2D mice were validated using clinical blood chemistry. These effects were not secondary to the neuromuscular phenotype, as determined by comparison with another, genetically unrelated mouse strain with similar neuromuscular dysfunction. However, these changes do not seem to be causative or consistent metabolites of CMT2D, because they were not observed in a second mouse *Gars* allele or in serum samples from CMT2D patients. Therefore, the metabolite ‘fingerprint’ we have identified for CMT2D improves our understanding of cellular biochemical changes associated with *GARS* mutations, but identification of efficacious treatment strategies and elucidation of the disease mechanism will require additional studies.

## Abstract (precis)

**Summary:** A metabolomics analysis of a mouse model of Charcot–Marie–Tooth type 2D neuropathy revealed a clear distinction between mutant and control samples, and the therapeutic potential of a subset of these changes was explored.

## INTRODUCTION

Charcot–Marie–Tooth disease (CMT) comprises a heterogeneous class of hereditary sensory and motor neuropathies caused by genetic defects in as many as 80 different loci in the human genome (Timmerman et al., 2014). The diseases can be broadly classified into Type 1 demyelinating neuropathies (CMT1) that result in reduced nerve conduction velocities, and Type 2 axonal CMTs (CMT2) that result in degeneration of peripheral motor and sensory axons. Type 1 CMTs typically arise from mutations in genes expressed by Schwann cells, the myelinating glial cells of the peripheral nervous system that predominantly encode proteins involved in myelin formation or stability. Type 2 CMTs are designated as axonal because the pathology arises directly in the motor and sensory axons. The mechanism(s) underlying axonal CMTs is much less clear than for the type 1 forms, but several forms of axonal CMT are associated with mutations in tRNA synthetase genes (aminoacyl-tRNA synthetases, or ARSs). These include glycyl-, tyrosyl-, alanyl-, and histidyl-tRNA synthetase (*GARS*, *YARS*, *AARS* and *HARS*), and more tentatively, methionyl- and lysyl-tRNA synthetase (*MARS* and *KARS*) (Antonellis et al., 2003; Jordanova et al., 2006; Latour et al., 2010; McLaughlin et al., 2010; Scheper et al., 2007; Vester et al., 2013).

The link between these ARSs and peripheral neuropathy suggests a shared pathogenic mechanism, and a straightforward loss of function has been proposed (Antonellis and Green, 2008; Griffin et al., 2014). However; tRNA synthetases are ubiquitously expressed, and each serves the indispensable and non-redundant function in protein synthesis by charging amino acids onto their cognate tRNAs. This function is strongly conserved through evolution, and why dysfunction in this activity would specifically lead to degeneration of peripheral axons is unclear (Motley et al., 2010; Park et al., 2008; Schimmel, 2008). Alternatively, gain-of-function mechanisms related to inhibition of VEGF/neuropilin1 signaling during development and inhibition of translation, independent of changes in tRNA charging, have also been reported for mutant forms of GARS (He et al., 2015; Niehues et al., 2015). We have begun to investigate possible disease mechanisms and pathogenic pathways using a metabolomics analysis in a mouse model of Charcot–Marie–Tooth type 2D (CMT2D), caused by a mutation in glycyl-tRNA synthetase (*Gars^Nmf249/+^*, MGI:3513831). Mice with dominant mutations in *Gars* develop peripheral neuropathy beginning by two weeks of age (Seburn et al., 2006). These mice have weakness and muscle atrophy, denervation at neuromuscular junctions that worsens in distal muscles, a decrease in axon diameters, and a reduction in the number of motor and sensory axons in the periphery (Seburn et al., 2006; Sleigh et al., 2014). They are, therefore, a genetically and phenotypically accurate model of CMT2D, with both face validity and construct validity, although the severity and early onset of their phenotype are worse than typically observed in CMT2D patients. A milder phenotype is found in *Gars^C201R^*^/+^ mice, which may be more representative of most patients. Neither mutation precisely reproduces a human disease-associated variant, but both share genetic and phenotypic characteristics of CMT2D.

We collected affected tissues (spinal cord and sciatic nerve) from the severe allele, *Gars^Nmf249/+^,* and wild-type littermate control mice at 6 weeks of age (four weeks post-onset) for metabolite profiling by mass spectrometry (metabolomics analysis). The severe allele was chosen to maximize the likelihood of finding changes in this first-of-its-type experiment. From these data, we have generated a definitive ‘fingerprint’ of changes in metabolite levels that define the differences between wild-type and mutant tissue. Furthermore, we have explored the possibility of using results from this analysis as biomarkers of CMT2D, and tested disease mechanisms and treatment strategies suggested by the data. Our long-term goal in these studies, and our rationale for using affected tissues instead of easily obtainable serum or urine samples, is to determine the mechanism by which mutations in *Gars* cause peripheral neuropathy, which should lead to treatment options based either on supplementation or drug interventions in the affected metabolic pathway. This determination will require additional comparisons, including comparisons to *Gars* mutations at different time points and to other neuropathy models; however, these results provide an excellent starting point for such studies, and an interesting point of comparison for metabolomics studies on other related diseases as such data becomes available.

## RESULTS

### Metabolite profiling of *Gars^Nmf249/+^* mice

Spinal cords and sciatic nerves were collected from 10 *Gars^Nmf249/+^* and 12 wild-type littermate controls at six weeks of age, approximately four weeks after the onset of the mutant phenotype (see Materials and Methods). Importantly, no immune infiltration or cell death is seen in the mutant spinal cord at this age (Seburn et al., 2006). These samples were used for metabolomics analysis, performed at Metabolon, Inc. (http://www.metabolon.com), in an attempt to identify changes in metabolite abundance that may be indicative of the pathophysiology underlying CMT2D. For spinal cords, two mutant samples had low mass and were therefore pooled with other samples for a total of eight independent replicates. The sciatic nerves were pooled into one mutant sample and one control sample due to the small size of the tissue. Therefore, all statistical analyses described were performed on the spinal cords, and sciatic nerves were simply assessed as agreeing or disagreeing with results in the spinal cord.

**Fig. 1.**
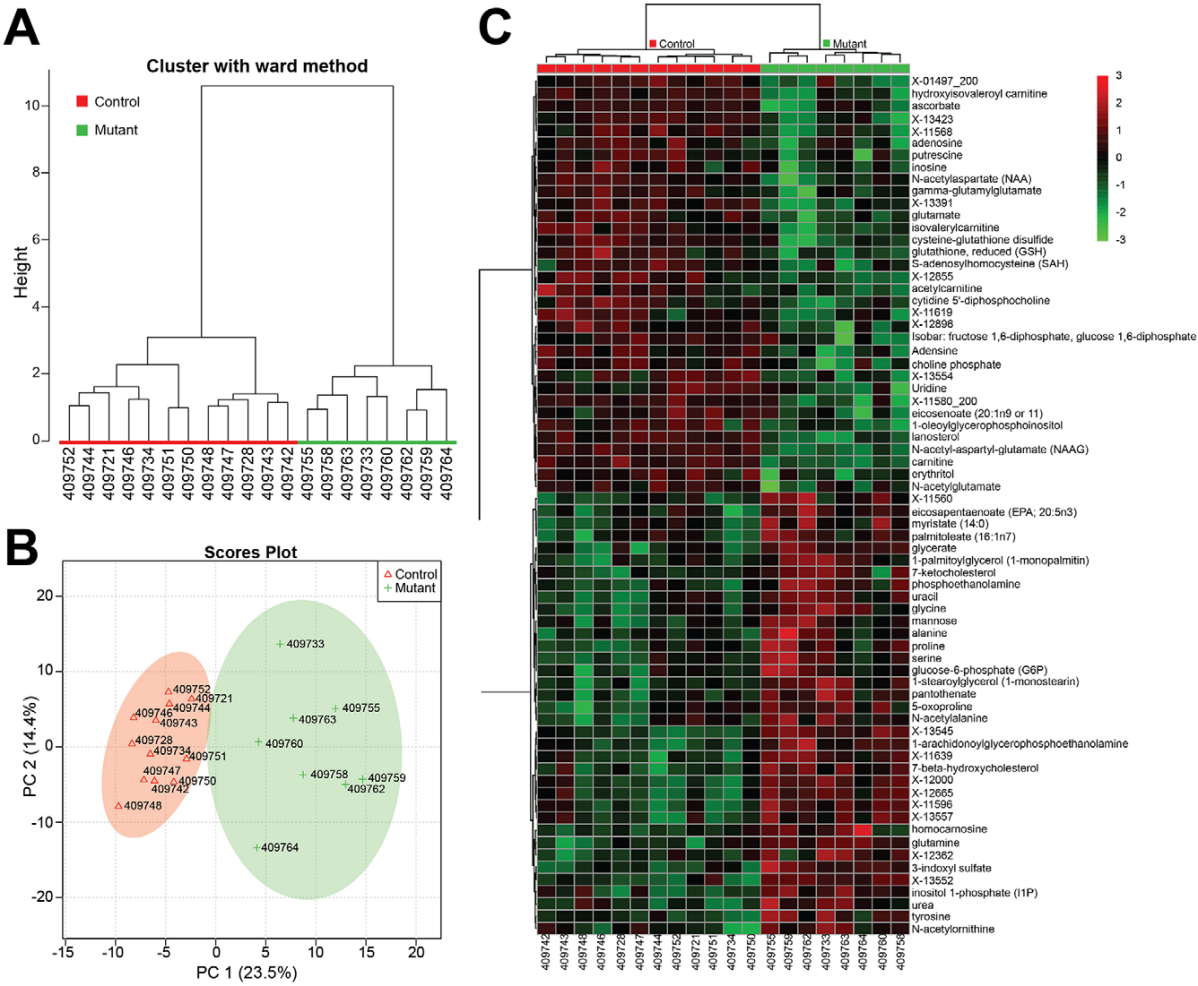
**Statistical analyses of metabolomics results separate *Gars^Nmf249/+^*/CMT2D samples from littermate controls.** (A) A hierarchical clustering analysis separates the mutant and control samples into two distinct clades. (B) Principal component analysis also separates the samples by genotype when plotted against the first two principal components. (C) A heat map of the top 70 most significant metabolites from the Student's *t*-test analysis also distinguishes mutant and control samples. Metabolites names are abbreviated, but full names are provided in Table S1. Metabolite names beginning with X- are detected metabolites based on mass and retention time, but are of unknown chemical structure.

In the spinal cord tissue, our exploratory analysis showed a clear separation between the mutant and control samples. The mutant and control samples separated in two different clades in a hierarchical clustering analysis (Fig. 1A). A principal component analysis (PCA) also showed clear separation between the mutant and control samples (Fig. 1B). A heat map of the top 70 metabolites, which were selected using Student's *t*-test, also shows clear separation between the two genotypes (Fig. 1C; see Table S1 for a full results of *t*-test analysis).

**Fig. 2.**
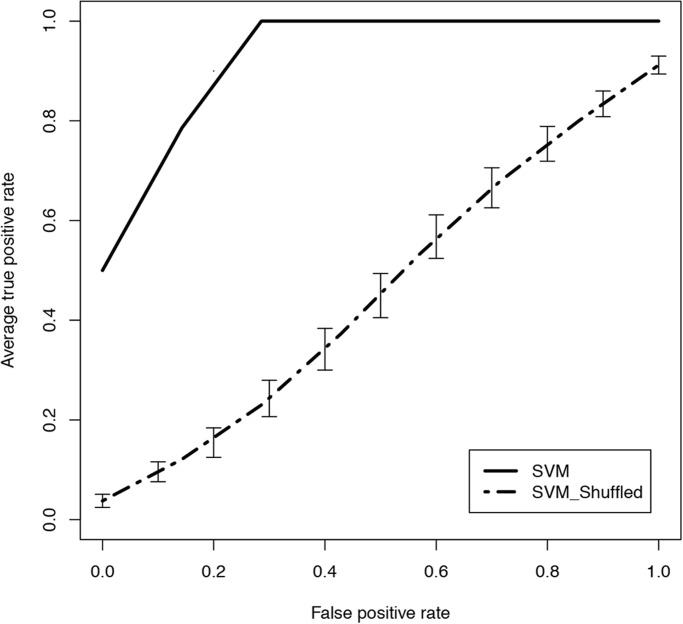
**ROC curve performance of the Support Vector Machine (SVM) classification models of mutant versus control samples.** ROC curve performance of the classification models from 50 iterations of the training and validation sets showing a perfect classification (solid line). The modeling process was repeated with random permutations of the diagnosis class labels, which showed near random classification (dashed line). This suggests that the model classification accuracies were not random results and the data contains valid biological signal. Vertical bars on the random set represent the standard error of the mean.

To establish which metabolites best distinguish the mutant and control samples, a support vector machine (SVM) classification (Vapnik, 1995) was performed using a nested cross validation approach. Fifty resampled iterations of the training and test samples were created from the control and mutant samples and the SVM models were trained on the training set and tested on the corresponding test set. The results showed robust discrimination between the affected and unaffected tissues (AUC=1), as compared to the 50 resampled iteration of the SVM classifier on samples with randomly permuted class labels (AUC=0.45, 95% CI 0.39-0.51). The receiver operating characteristic (ROC) curve derived from averaging the performance of the 50 resampled iterations shows that the difference seen between the two genotypes has a true biological signal (Fig. 2). A metabolite ‘fingerprint’ that discriminates between mutant and control samples was derived by average rank of the top metabolic features of the 50 SVM iterations analysis to determine the most significant and robust changes. The top 25 distinguishing metabolites, as defined by this analysis, are shown in Table 1, along with their original *t*-test *P*-values, false discovery rate, and fold-change (log_2_). The metabolites that consistently appear as important for the discrimination of the two genotypes in 50 iterations of the SVM algorithm are potential biomarkers, based on their differential abundance in mutant versus control tissue.

**Table 1.**
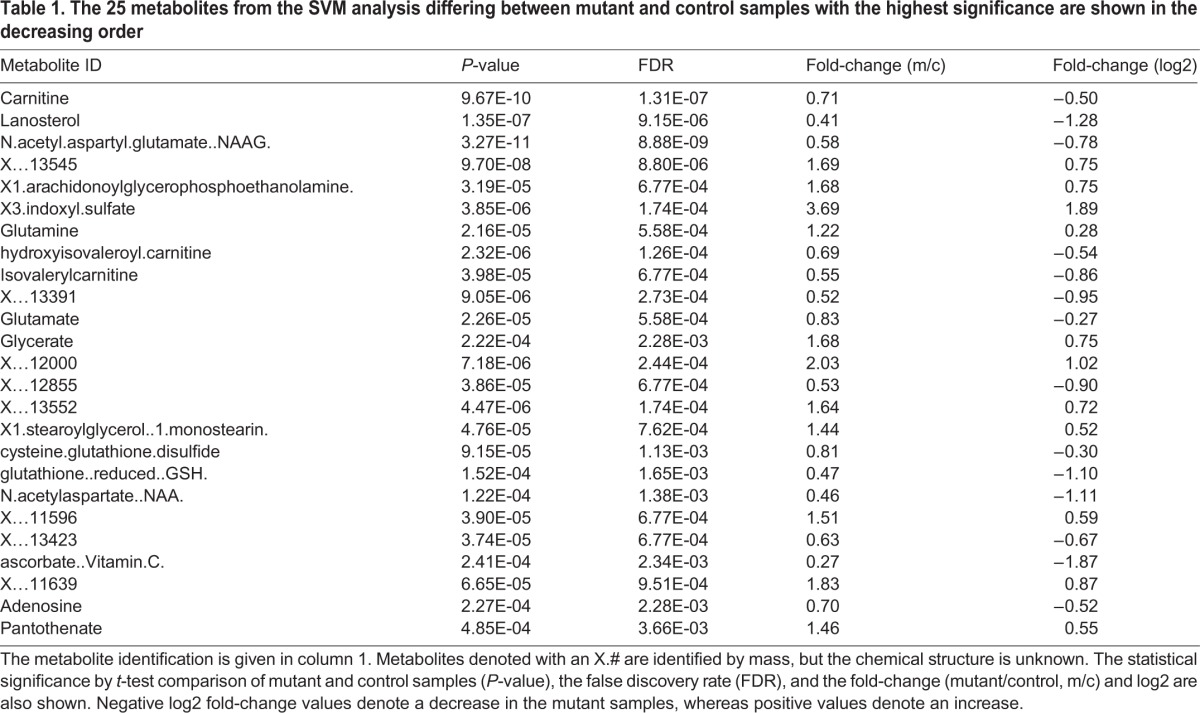
**The 25 metabolites from the SVM analysis differing between mutant and control samples with the highest significance are shown in the decreasing order**

In the *t*-test analysis, 112 metabolites showed statistically significant changes between the two genotypes (*P*-value≤0.05). Results from the pooled sciatic nerve samples generally agreed with the results from spinal cord (Table S2). Changes were more consistent for those metabolites that increased in mutant samples, but the metabolites with the greatest magnitude of decrease in spinal cord were also decreased in sciatic nerve.

The metabolites with the largest magnitude decrease in the mutant samples included ascorbic acid (0.27 mut/cont, *P*=2.4×10^−4^) and carnitine (0.71 mut/cont, *P*=9.7×10^−10^), whereas glycine showed a modest increase (1.14 mut/cont, *P*=6.5×10^−4^). These metabolites are potential targets for therapy through dietary supplementation. Ascorbic acid has been implicated as a possible therapeutic in demyelinating forms of CMT (Pareyson et al., 2006), but has not been previously associated with axonal neuropathy. Carnitine supplementation has been suggested to be beneficial in a variety of neurological settings in both human and animal studies, including the regeneration of peripheral axons after injury (Chan et al., 2014; Chiechio et al., 2007; Hart et al., 2002; Karsidag et al., 2012). Interestingly, carnitine and its derivatives were decreased in spinal cord, but were consistently increased in sciatic nerve. Glycine has many functions such as neurotransmission and folate metabolism, but it is also the direct substrate for GARS, and a partial loss of enzymatic function may lead to substrate accumulation and may be remedied by increasing substrate concentration. However, the protein product of the *Gars^Nmf249^* allele (*GARS* P278KY) is enzymatically active in assays using recombinant protein (Seburn et al., 2006). Glycine is involved in many metabolic pathways besides translation, and although statistically significant, the increase is only 1.14-fold, ranking it at 53rd in importance from the SVM analysis. Ascorbic acid, carnitine and various carnitine derivatives were listed as the top metabolites from the 50 iterations of the SVM analysis (Table 1). In addition to these three metabolites described above, levels of several metabolites in the cholesterol and neurotransmitter biosynthetic pathways, and all metabolites associated with the urea cycle, were elevated in the *Gars^Nmf249/+^* samples. We performed preliminary follow up studies on the metabolomics results for ascorbic acid, carnitine, glycine, and for markers of possible liver/kidney dysfunction.

### Ascorbic acid does not provide a serum biomarker for CMT2D

Ascorbic acid had one of the largest changes in magnitude in our study, with a 75% reduction in the *Gars^Nmf249/+^* spinal cord. Ascorbic acid supplementation has been investigated as a treatment for demyelinating CMT1A based on success in transgenic animal models overexpressing *Pmp22*, the genetic cause of CMT1A (Pareyson et al., 2006; Passage et al., 2004). However, the connection of ascorbic acid to axonal neuropathy is unclear. We examined serum ascorbate levels to determine if the changes observed in the spinal cord were systemic, and if ascorbic acid may be useful as a biomarker of CMT2D.

Serum from four *Gars^Nmf249/+^* mice at four weeks of age was tested for ascorbic acid levels using a colorimetric assay (see Materials and Methods). These mice were compared to six wild-type littermates and to three mice carrying a milder allele of *Gars* (*Gars^C201R/+^*) (Achilli et al., 2009). No consistent differences in serum ascorbic acid levels were observed. The *Gars^Nmf249/+^* mice had 104±40 µM (mean±s.d.) ascorbate, levels higher than littermate controls (80±16 µM), but not significantly so (*P*=0.17, Student's *t*-test). In contrast, mice with the milder *Gars^C201R/+^* mutation had reduced serum ascorbate (53±11 µM, *P*=0.04, Student's *t*-test. Note: these mice were 8 weeks of age vs 4 weeks for the previous comparison). These values are comparable to published ascorbic acid levels obtained through different quantitative methods and appear to be internally consistent, but do not approach the fourfold change observed in spinal cord (Cherdyntseva et al., 2013; Furusawa et al., 2008; Li et al., 2008). Thus, changes in serum ascorbate do not provide a reliable indicator of ascorbate levels in the spinal cord, suggesting that the changes observed by mass spec in spinal cord are not systemic.

### Glycine supplementation does not alter neuropathy

**Fig. 3.**
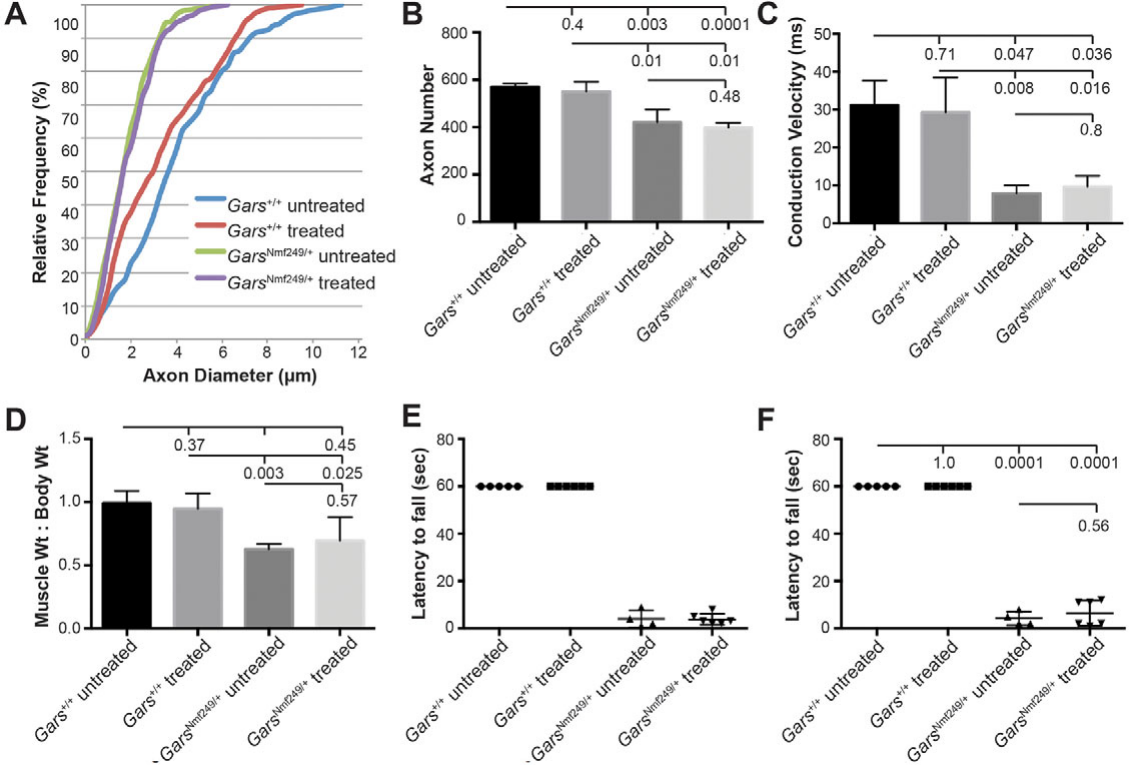
**Glycine supplementation does not improve neuropathy.** (A) A cumulative histogram of axon diameters in the motor branch of the femoral nerve from treated and untreated *Gars^Nmf249/+^* mice and littermate controls shows the distribution of axon diameters in *Gars* mutant mice does not change with glycine supplementation (*P*=0.9, K–S test). In wild-type mice, glycine supplementation was not detrimental (*P*=0.28, K–S test). (B) Axon number in the motor branch of the femoral nerve was not changed with glycine supplementation, both treated and untreated mutant nerves had reduced axon number compared to controls (*P*≤0.01), but control treated nerves did not have altered axon number compared to untreated controls (*P*=0.39), and mutant treated nerves were not different from untreated mutant nerves (*P*=0.48). (C) Nerve conduction velocity was also unchanged by glycine supplementation. Both treated and untreated mutant sciatic nerves conducted more slowly than control littermates (*P*<0.05), whereas treated controls were the same as untreated controls (*P*=0.71) and treated mutants were the same as untreated (*P*=0.82). (D) Muscle weight:body weight ratio, an indicator of muscle atrophy, was unchanged with glycine supplementation. Mutant muscles of the triceps surae showed reduce mass indicative of atrophy with glycine supplementation (*P*<0.050), whereas control muscles were unchanged with glycine supplementation (*P*=0.37) as were mutant muscles (*P*=0.57). Untreated control and treated mutant muscles were not significantly different owing to the variability and small sample size. Error bars represent standard deviation from the mean. (E,F) The wire hang test of grip strength and endurance at 3.5 weeks of age (E) and 5 weeks of age (F) revealed that control mice were able to complete the test, hanging on for one minute with and without glycine supplementation, whereas mutant mice were able to hang for <10 s with or without glycine supplementation. No improvement with glycine treatment in the mutant animals was seen at either age (*P*=0.56). *N*=6 WT treated and *N*=3 untreated, *N*=4 *Gars^Nmf249/+^* treated and *N*=6 untreated.

The modest elevation in glycine observed in the spinal cord of *Gars^Nmf249/+^* mice (1.14-fold increase over control) could be consistent with an elevation in the reaction substrate if there is a loss of enzymatic activity in mutant GARS. We therefore tested whether further increasing substrate levels through glycine supplementation could counteract such a mechanism. Glycine was added to a soft diet provided beginning at 2 weeks of age for four mutant and six littermate control mice, and the neuropathy and motor performance of these mice was compared to six mutant and three wild-type littermate control mice on the same diet without glycine supplementation. Mice were group housed, so food consumption by individual mice is unknown; however, overall food consumption was measured by weight three times per week and did not differ between groups, indicating that glycine did not cause an aversive taste reaction, for example. At five weeks of age, the glycine supplementation did not improve axon size (Fig. 3A) or number (Fig. 3B). Consistent with the unaltered neuropathy, nerve conduction velocity and muscle atrophy, assessed by the ratio of muscle weight to total body weight, were also unchanged (Fig. 3C,D). Gross motor performance was assessed with a test of grip strength and endurance, the wire hang test, in which mice are placed on a wire grid that is then inverted, and the latency to fall (up to one minute) is recorded (see Materials and Methods). Mice were tested longitudinally at 3.5 weeks (Fig. 3E) and 5 weeks of age (Fig. 3F), and no improvement with glycine supplementation was seen at either age. Although this was a pilot study, no indication of positive effects was observed, and testing in additional animals was not pursued. Since glycine is involved in many metabolic and physiological pathways, the lack of effect may indicate that the elevation seen in our metabolomics analysis is not related to loss of function in tRNA charging. Alternatively, if a loss of function is associated with the *Nmf249* allele, it may not be responsive to an increase in substrate concentration. Although glycine supplementation did not show adverse effects in control mice, it does not appear to be an efficacious treatment option, at least for the *Gars^Nmf249^* allele of *Gars*.

### Carnitine supplementation improves motor performance, but does not alter neuropathy

**Fig. 4.**
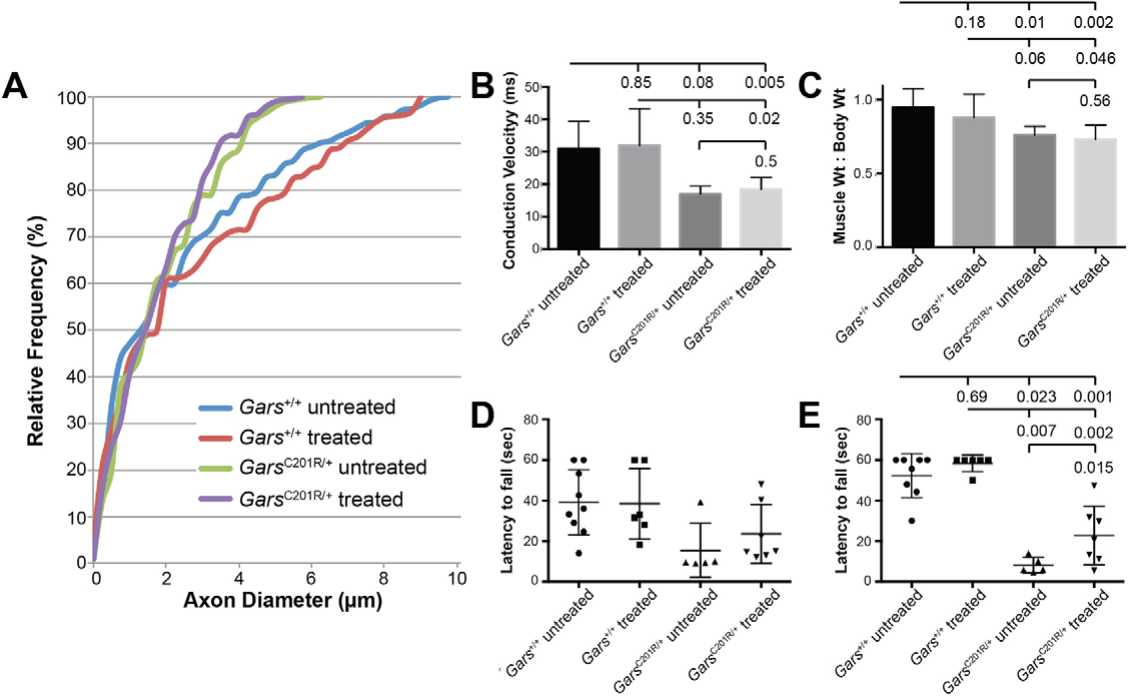
**Carnitine supplementation improves motor performance, but does not alter neuropathy.** (A) A cumulative histogram of femoral motor nerve axon diameters in the milder *Gars^C201R/+^* model of CMT2D shows that supplementation with acetyl-L-carnitine does not improve axon atrophy. Both treated and untreated mutant axons are smaller than control (*P*<0.05, K–S test). Carnitine supplementation had no effect on control axon diameters (*P*=0.51), and mutant axons were also unchanged (*P*=0.21). (B) Nerve conduction velocity (NCV) in the sciatic nerve was also unchanged. Mutant axons with or without carntine supplementation conducted more slowly than untreated control axons (*P*<0.05), control NCVs were unchanged by carnitine supplementation (*P*=0.85), as were mutant NCVs (*P*=0.50). Treated controls and untreated mutants were not statistically different owing to variability in the control values and small sample size. (C) Muscle weight:body weight ratio indicated atrophy in mutant triceps surea with or without carntine supplementation (*P*<0.05, WT treated versus mutant untreated, *P*=0.06). The control weights were unchanged by carnitine supplementation (*P*=0.18), as were the mutants (*P*=0.56). Error bars represent standard deviation from the mean. (D,E) Supplementation with acetyl-L-carnitine did improve motor performance in the wire hang test of grip strength and endurance. Results are shown at the beginning of treatment (3.5 weeks of age, D), and at 7.5 weeks of age (E). Although still worse than control (*P*<0.01), the treated *Gars^C201R/+^* mice did perform better than untreated mutant mice (*P*=0.015). *N*=6 WT treated and *N*=7 untreated; *N*=9 *Gars^C201R/+^* treated and *N*=5 untreated. All measures at 9 weeks of age except wire hang, which was performed at 3.5 and 7.5 weeks of age.

The decrease in carnitine and related derivatives observed in the *Gars^Nmf249/+^* spinal cord samples (from 55% to 83% of control, average change 72%) also suggested a possible target for treatment by dietary supplementation. Carnitine facilitates mitochondrial function by mediating transport of fatty acids into the mitochondria for metabolism (Fritz et al., 1958). In addition, carnitine and its derivatives have been suggested to promote peripheral nerve regeneration (Callander et al., 2014; Chan et al., 2014; Chiechio et al., 2007; Hart et al., 2002; Karsidag et al., 2012). To test whether carnitine supplementation would improve or reverse the symptoms of neuropathy observed, we added acetyl-L-carnitine, a more bioavailable form (Liu et al., 2004), to the drinking water of mice at 1% w:v based on previous studies in rats (Hagen et al., 2002; Liu et al., 2002a,b). Given our lack of success with glycine supplementation, and to test whether our results would generalize to other alleles of *Gars*, we performed this study on mice carrying a milder *Gars^C201R^* variant (Achilli et al., 2009). In total, nine mutant and six littermate control mice were supplemented with acetyl-L-carnitine, and results were compared to five mutant and seven littermate control mice that did not receive supplementation. Carnitine supplemented water was the only water source for the duration of the experiment (weaning at 3.5 weeks of age to 9 weeks of age). Again, mice were group housed so consumption by individuals is unknown, but overall water consumption did not differ between groups. Axon atrophy was not improved with treatment (Fig. 4A), and mice with the milder *Gars^C201R/+^* allele do not have a reduction in axon number compared to control (Achilli et al., 2009; Motley et al., 2011), so this was not measured. Nerve conduction velocity and muscle atrophy were also not improved by acetyl-L-carnitine supplementation (Fig. 4B,C). However, gross motor performance in the wire hang test was improved with acetyl-L-carnitine supplementation compared to untreated *Gars ^C201R/+^* mice, with the effect most significant at 8 weeks of age (Fig. 4D,E, *P*=0.015), although the treated mice still performed far worse than wild-type littermate controls (*P*<0.01). Given the lack of improvement in axon size and nerve conduction velocity, the improvement in the wire hang test does not reflect an improvement in the neuropathy itself, and effects may be in muscle or other factors.

### Liver and kidney dysfunction in *Gars^Nmf249/+^* mice, but not other CMT2D mice or patients

**Fig. 5.**
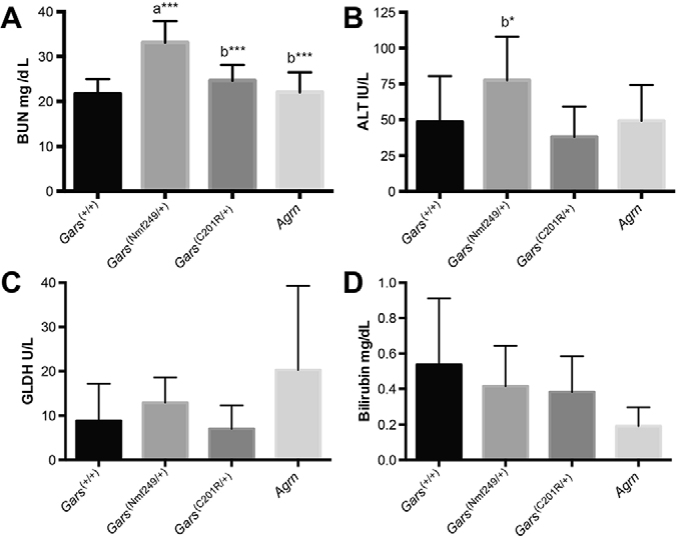
**Serum indicators of liver and kidney function in neuromuscular disease model mice.** (A) Blood urea nitrogen (BUN), an indicator of kidney function, is elevated *Gars^Nmf249/+^* mice, but not in the milder *Gars^C201R/+^* allele, nor in *Agrn^nmf380^* mice, a model of congenital myasthenic syndrome that causes severe neuromuscular dysfunction that is comparable to the *Gars^Nmf249/+^* mice. Both the milder *Gars* mice and the *Agrn* mutant mice had significantly lower BUN levels than the severe *Gars* mice. (B) The liver enzyme alanine transaminase (ALT) was also elevated in *Gars^Nmf249/+^* mice compared to the milder *Gars^C201R/+^* mice, but neither *Gars^C201R/+^* nor in *Agrn* mutant mice differ from control. (C,D) Other indicators of liver function, glutamate dehydrogenase (GLDH) and total bilirubin were highly variable in all gentoypes and did not show significant changes. *N*=11 wild-type, *N*=8 *Gars^Nmf249/+^*, *N*=5 *Gars^C201R/+^*, and *N*=6 *Agrn* mice. ‘a’=different than control, ‘b’=different than *Gars^Nmf249/+^* *P≤0.05, **P≤0.01, ***P≤0.001. Error bars represent standard deviation from mean.

Our initial metabolite profile also suggested a level of liver and kidney dysfunction in the *Gars^Nmf249/+^* mice. For example, all metabolites associated with the urea cycle were elevated in the mutant mice. To further explore this possibility and to determine if liver and kidney dysfunction could be a primary cause of the neuropathy phenotype, blood urea nitrogen (BUN), the liver enzymes glutamate dehydrogenase (GLDH) and alanine transaminase (ALT), and total bilirubin were tested in serum samples from four-week-old *Gars^Nmf249/+^* mice and littermate controls (Fig. 5). Consistent with the conclusions of the mass spec-based analysis, BUN and ALT were significantly elevated in the *Gars^Nmf249/+^* mice. Bilirubin and GLDH were not significantly changed, but values were extremely variable in all genotypes.

The *Gars^Nmf249/+^* mice are smaller than littermates (approximately 35% reduction in body weight) and have compromised neuromuscular performance even at four weeks of age. It is therefore possible that the serum changes in BUN and ALT are the result of secondary consequences such as malnutrition or dehydration, although the mice used in these studies were weaned for less than one week before testing. To control for this possibility, we again examined the milder *Gars^C201R/+^* mice at 8 weeks of age. These mice are only slightly smaller than control littermates (approximately 10% reduction in body weight) and are difficult to distinguish based on overt neuromuscular performance. These mice failed to show indications of liver and kidney dysfunction, and values were more similar to control than to *Gars^Nmf249/+^*.

As a final comparison, we also tested serum from an independent neuromuscular mutation. This mutation is a recessive, single amino acid change in *Agrn* (*Agrn^Nmf380^*, MGI:3614578), encoding a heparan sulfate proteoglycan critically involved in neuromuscular junction formation in mice. Human mutations in *AGRN* cause a congenital myasthenic syndrome that closely resembles phenotype of the mouse point mutation (Bogdanik and Burgess, 2011; Huze et al., 2009; Maselli et al., 2011). The *Agrn* homozygous mice are similarly runty and also have impaired neuromuscular function. Therefore, if symptoms of liver and kidney dysfunction observed in the *Gars^Nmf249/+^* mice are secondary to this condition, we would anticipate similar changes in the *Agrn* mice. However, BUN, ALT, and GLDH were not different between *Agrn* mutant mice and littermate controls or *Gars^+/+^* control values (shown), suggesting that these changes are specific to the *Gars^Nmf249/+^* mutant mice and not secondary to impaired neuromuscular performance.

To explore liver and kidney dysfunction as a possible disease mechanism or complication in CMT2D patients, clinical blood chemistries were examined in ten patients carrying the *GARS^L129P^* allele (Table 2). Patients were diagnosed with motor and sensory neuropathy based on clinical evaluation and electrophysiology, and were known carriers of the *GARS^L129P^* mutation. However, blood chemistries for these patients were normal with few values falling outside the normal range (Table 2). The lack of elevated uric acid, creatinine and urea, together with the absence of clinical signs and complaints, strongly excludes insufficiency of kidney function in all patients tested. Although albumin levels are slightly above normal values in some patients, the normal values for ALT (alanine transaminase) and AST, together with the lack of clinical signs and no anaemnestic data for hepatitis excludes liver dysfunction. The slight increases alkaline phosphatase or billirubin in some patients also do not support liver insufficiency or any defects in the hepatocytes, but could be an indication of a problem in the bile ducts. Therefore, liver and kidney dysfunction do not seem to be hallmarks of CMT2D, despite positive results in both metabolomics analysis and serum chemistry in the *Gars^Nmf249/+^* mice.

**Table 2.**
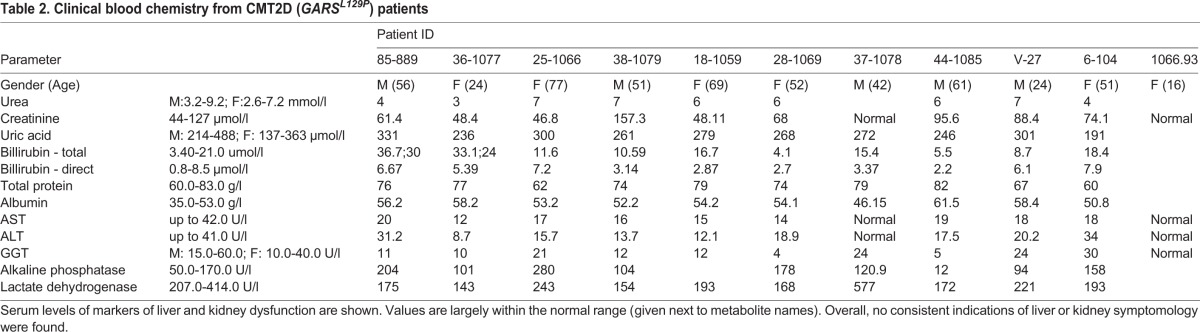
**Clinical blood chemistry from CMT2D (*GARS^L129P^*) patients**

## DISCUSSION

The metabolomics analysis of spinal cord from a mouse model of CMT2D revealed a distinct metabolite fingerprint associated with the disease genotype in *Gars^Nmf249/+^ mice*. In a total of 272 metabolites conclusively identified by mass spectrometry, 56 were significantly elevated and 56 were significantly decreased in the *Gars^Nmf249/+^* mice. These numbers, and the fact that metabolites were observed to change in both directions is consistent with results from a study on amyotrophic lateral sclerosis (ALS) (Rozen et al., 2005). Although we identified a number of significant differences in the metabolite profiles of mutant mice versus controls, determining which differences are direct indicators of the disease mechanism and which are secondary to the compromised neuromuscular function of these mice will require additional comparisons. Informative comparisons would include tissue from *Gars^Nmf249/+^* mice at additional time points during disease progression (particularly pre-onset), an equivalent analysis on *Gars* mutant mice that have milder phenotypes (i.e. *Gars^C201R/+^* mice), as well as other phenotypically similar neuromuscular disease models, such as *Nefl* mutant mice, a model of CMT2E (Adebola et al., 2015; Zhu et al., 1997).

In a study of acute peripheral nerve injury caused by ligation of the spinal nerve in rat, a metabolomic profile using serum indicated stress responses such as increased norepinephrine (Mao et al., 2009). Similar changes were not observed in the *Gars^Nmf249/+^*, distinguishing the chronic neuropathy from acute injury. In spinal cord tissue from the CMT mice, ascorbic acid was reduced by 75%, one of the largest magnitude changes. Vitamin C is known to promote myelination and had positive effects *in vivo* in a mouse model of CMT1A (Passage et al., 2004); however, reports on the effectiveness of this treatment in patients are largely negative (Burns et al., 2009; Micallef et al., 2009; Pareyson et al., 2011; Verhamme et al., 2009)**.** The mechanism through which ascorbic acid would relate to axonal neuropathy is unclear.

In our axonal CMT2D model, the decrease in ascorbic acid was observed in spinal cord, but was not seen in serum. This result has several interesting implications. First, ascorbic acid may not represent a good biomarker of CMT2D, because it is not altered in easily sampled sources such as serum. In addition, the fact that ascorbate levels are changed in the affected tissue and not the serum may indicate that this change is more directly related to the disease mechanism and not a secondary systemic or dietary change. Identifying such differences highlights an advantage of metabolomic studies on animal models, in which affected tissues may be analyzed directly. The lack of change in serum ascorbate also suggests that vitamin C supplementation may not be effective for CMT2D, since circulating levels are not reduced and bioavailability or uptake into the spinal cord may also be a factor. Finally, ascorbate also illustrates a challenge to analyzing metabolomics data from mouse models using existing pathway annotations, which are largely derived from human data. Mice are able to generate ascorbic acid, whereas humans are dependent on dietary Vitamin C. Therefore, changes in ascorbic acid levels in the spinal cord of the CMT2D mouse model may have different mechanistic significance and merit further investigation.

Supplementation with either glycine or acetyl-L-carnitine did not produce promising results in terms of correcting primary measures of neuropathy such as axon size, axon number, or nerve conduction velocity. Carnitine supplementation was modestly beneficial in gross motor performance as assayed in the wire hang test. Given the lack of positive outcomes in the neuropathy measures, this improvement does not reflect a slowing or reversal of the disease itself. Both the glycine and carnitine studies were modestly powered statistically, but as pilots, they did not provide any promising outcomes that would justify larger study cohorts.

There are many possible reasons for the lack of efficacy with these interventions. First, the changes in metabolite levels may be secondary to the disease process and not causative, in which case attempting to restore normal levels may be of little benefit. This seems likely in the case of carnitine and its derivatives, which are generally associated with mitochondrial function and general cellular metabolism. The increase in glycine was modest, and was tested in the context of a possible loss of function leading to increased enzymatic substrate. However, glycine is involved in many cellular processes including synaptic transmission in the spinal cord. Thus, the changes in glycine levels in the *Gars*^Nmf249/+^ mice may be due to changes in pathways other than tRNA charging. Alternatively, the P278KY mutation in GARS may cause a defect that is not remedied by increasing substrate concentration. Finally, the 1.14-fold increase in glycine levels may be statistically significant, but may not be of any biological consequence, and we did not confirm increases in glycine levels in tissues of the mice receiving the supplemented diet. Therefore, while supplementation with glycine or carnitine would have presented a safe and inexpensive therapeutic strategy, the effectiveness of this approach is not supported by our data.

In addition to identifying possible therapeutic interventions, the potential promise of using metabolite profiling to understand disease mechanisms including neuropathies is demonstrated by recent work on serine palmitoyltransferase long-chain base subunits 1 and 2 (SPTLC1 and 2), a heterodimeric enzyme that links palmitoylate onto serine at an early step in complex sphingolipid biosynthesis. Mutations in these genes cause hereditary sensory and autonomic neuropathy 1 (HSAN1) (Dawkins et al., 2001; Rotthier et al., 2010). As a result of altered substrate specificity in the enzyme, alanine or glycine are placed onto the palmitoylate in place of serine, creating a pathological gain-of-function and leading to the production of toxic deoxysphingoid bases that promote axonal degeneration *in vitro*. These novel deoxysphingoid bases are detectable in serum and tissue of both HSAN1 patients and a transgenic mouse model (Eichler et al., 2009; Penno et al., 2010). Therefore, profiling metabolite changes in HSAN1 contributed directly to understanding the disease mechanism, although these changes were not initially found in a high throughput mass spectrometry-based approach.

While it is attractive to draw an analogy between HSAN1 and CMT2D, the profile of metabolic changes in the *Gars^Nmf249/+^* mouse spinal cord did not highlight a specific disease process, but generally suggested liver and kidney dysfunction. Liver pathology has been associated with recessive mutations in other tRNA synthetase genes (Casey et al., 2012; Sofou et al., 2015). The liver dysfunction in *Gars^Nmf249/+^* mice was supported by clinical blood chemistries in the mouse, and such changes were not present in mice with another neuromuscular mutation, suggesting that the changes are specific to the *Gars* mutations and not secondary to impaired neuromuscular ability. Analysis of CMT2D patients and the milder *Gars^C201R/+^* mouse model did not support the conclusion that liver and kidney dysfunction is a primary or causative feature of the disease. This result could arise because traditional biomarkers of liver and kidney dysfunction used in clinical assessment are less sensitive indicators than other metabolites (Cassol et al., 2013; Shlomai et al., 2013), because of species or allelic differences between the *Gars^Nmf249/+^* mice and CMT2D patients, because the severity of the *Gars^Nmf249/+^* mice more closely mimics the homozygous state reported for human tRNA synthetase mutations with liver dysfunction, or because supportive care for patients eliminates these aspects of the disease. Nonetheless, these results do merit consideration based on the significant and specific effects in the *Gars^Nmf249/+^* mice.

Other interesting and noteworthy differences in metabolite levels include a large number of amino acids that are changed, with most being elevated in mutant samples. Amino acids are involved in protein synthesis, but also serve as substrates and intermediates in many biochemical pathways and a clear theme did not emerge. Similarly, nucleoside and nucleotide levels were often altered, again suggesting possible differences in intracellular signaling pathways. Finally, antioxidants including ascorbic acid and forms of glutathione were reduced in mutant samples, suggesting changes in oxidative pathways, but inconsistent with the upregulation typically seen in response to oxidative stress.

The *Gars* point mutations are both autosomal ‘dominant’ mutations, in that mice with a mutant allele in combination with a wild-type allele display a neuropathy phenotype. However, both the mild *C201R* and the severe *Nmf249* allele fail to complement a presumed null allele that eliminates *Gars* expression at the mRNA level without producing a mutant protein, resulting in embryonic lethality. (Achilli et al., 2009; Seburn et al., 2006). This phenotype is inconsistent with a peripheral neuropathy, because a functional peripheral nervous system is not required until birth when the animal has to breathe independently. These results could be explained if the mutant forms of the protein are assuming a pathological function (neomorphs) that cause neuropathy even in the presence of a wild-type allele, but also fail to support their normal activity in charging glycine onto tRNA^Gly^ despite retained enzymatic activity of recombinant protein carrying the equivalent amino acid changes. An alternative explanation of the embryonic lethality is that other organ systems such as the liver are also affected by the mutations in the absence of wild-type compensation, resulting in the early developmental phenotype. Finally, transgenic overexpression of wild-type *GARS* completely rescues the embryonic lethality of both point mutations in combination with the null allele, but does nothing to correct the neuropathy, suggesting that there are loss of function aspects of the point mutations that impair viability, but that the neuropathy is the result of a pathological gain-of-function that the wild-type protein cannot out compete (Motley et al., 2011). Thus, allele specific changes may be a result of combined loss- and gain-of-function mechanisms.

Understanding the disease mechanisms underlying the puzzling association of tRNA synthetases and peripheral neuropathy will require a combination of genetic and biochemical approaches. Here we present a first metabolomic fingerprint from the spinal cord of a CMT2D mouse model. These data represent an important first step that provides a basis for future comparative studies in both mice and human populations and suggest carnitine supplementation as being potentially beneficial in treating CMT2D symptoms of weakness and fatigue, if not directly correcting the underlying neuropathy.

## MATERIALS AND METHODS

### Mice

All mice were maintained in the Research Animal Facility of The Jackson Laboratory under standard housing conditions including a 14:10 light:dark cycle and *ad libitum* food (NIH 6% chow) and water. All procedures were approved by the Animal Care and Use Committee of The Jackson Laboratory. All models have been previously described (Achilli et al., 2009; Bogdanik and Burgess, 2011; Seburn et al., 2006). Littermate animals were used as controls to avoid age- and genetic background-dependent effects. Mice of both sexes were used in each group. Tissues included spinal cord (>50 mg of tissue from each mouse) and sciatic nerve (5-10 mg of tissue per mouse). For metabolite profiling, individual spinal cords provided sufficient tissue for analyses with two exceptions that were pooled for a total of eight samples. Sciatic nerves were pooled into one *Gars^Nmf249/+^* and one *Gars^+/+^* wild-type sample.

### Human subjects

Patients participating in this study are part of a large Bulgarian family that contributed to the original identification of *GARS* mutations as the cause of CMT2D and its allelic disorder – distal spinal muscular atrophy type V (Antonellis et al., 2003; Christodoulou et al., 1995; Sivakumar et al., 2005). All patients provided informed consent and protocols and procedures were approved by the Institutional Review Boards of The Jackson Laboratory and Medical University-Sofia.

### Tissue collection

For spinal cord and sciatic nerve collection, six-week-old *Gars^Nmf249/+^* and *Gars^+/+^* littermate mice were asphyxiated by CO_2_ inhalation. The sciatic nerve of both thighs was quickly dissected free and snap-frozen in microfuge tubes in liquid nitrogen. The vertebral column from the sacral to the cervical vertebrae was then dissected free and opened with scissors along the dorsal aspect to expose the spinal cord. The spinal tissue was removed and similarly snap-frozen. Spinal cord dissection yielded 50-100 mg of tissue per animal, sciatic nerves were 5-10 mg. All tissue was stored at −70°C until shipping on dry ice for metabolomics studies.

### Glycine and acetyl-L carnitine supplementation

The normal mouse diet (5K20, Lab Diet) contains 0.94% glycine by weight with approximately 20% total protein by weight. To supplement glycine intake, we provide mice with Diet Gel 76A (Clear H2O) supplemented with 20 mg glycine/1 g in place of normal food and water. Diet gel is 76% water and 24% ‘food’, and 4.7% protein by weight, and thus approximately the same total protein content as the normal diet. This provides approximately five times the normal dietary glycine. Glycine was Ultra AjiPure pharmaceutical grade glycine powder (Swanson's Vitamins). Mice were provided the diet gel at two weeks of age, and this was their sole food and water source from three weeks of age (weaning) for the duration of the experiment (to five weeks of age). Control ‘untreated’ mice were provided diet gel with no added glycine. Acetyl-L carnitine was supplemented by dissolving in the drinking water at 1% w:v, thus dosing with approximately 30 mg/day based on average water consumption of 3 ml per mouse. The carnitine content of standard mouse diet is not specified, but as a grain-based diet, it is likely to be low, and 1% carnitine in drinking water should represent an increase of at least 100-fold in carnitine intake. These doses are similar to previous studies of carnitine supplementation in rats (Hagen et al., 2002; Liu et al., 2002a,b). Acetyl-L-carnitine has better bioavailability that unmodified carnitine, and was purchased as ALCAR acetyl-L-carnitine powder from Swanson's vitamins. Mice were provided the supplemented water from weaning (3.5 weeks of age) for the duration of the experiment (9 weeks of age).

### Assessment of neuropathy

The effects of glycine and acetyl-L-carnitine supplementation were assessed using a battery of measures that have been used to define the phenotype of the *Gars* mutant mice as valid models of CMT2D. Detailed methods are described in previous publications (Achilli et al., 2009; Burgess et al., 2010; Motley et al., 2011; Seburn et al., 2006) and are described in brief here. Nerve histology was performed on the motor branch of the femoral nerve. Nerves are dissected free and fixed in 2% glutaraldehyde, 2% paraformaldehyde in 0.1 M cacodylate buffer. Samples are then dehydrated through an alcohol series and plastic embedded. Nerves are sectioned at 500 nm and stained with Toluidine Blue. Photomicrographs are quantified for axon number and axon diameter using ImageJ (NIH). Nerve conduction velocities were determined by stimulating the sciatic nerve at sciatic notch (hip) and the ankle while recording muscle response in the hind paw. The distance between the distal and proximal sites of stimulation is divided by the difference in latencies to elicit a muscle action potential. These experiments are performed on mice anesthetized with isofluorane and maintained at a core body temperature of 37°C. As a measure of muscle atrophy, the triceps surae (consisting of the medial and lateral gastrocnemius, the soleus, and the plantaris) are dissected free from both hind limbs and weighed. This weight is compared to the total body weight to determine if there is disproportionate weight loss (atrophy) in muscles of the hindlimb. The wire hang test assesses grip strength and endurance. Mice are placed on a wire grid approximately 30 cm above a pen with bedding. The grid is then inverted, and the time to fall (s) is recorded. Three trials are performed with a maximum duration of 60 s per trial and a rest of 30 s between trials. Results presented the longest latency to fall among the three trials.

### Statistical analysis of phenotyping data

Differences in the distribution of axon diameters were tested using the Kologmorov–Smirnoff (K–S) test. All other measures (axon number, muscle weight:body weight, nerve conduction velocity, and wire hang test) were tested using a one-way ANOVA with Tukey's posthoc test. A *P* value≤0.05 was considered significant.

### Serum

Serum was collected by cardiac puncture from mice under isofluorane anesthesia. Whole blood was added to non-heparinized collection tubes and spun to obtain the serum fraction for use in ascorbate and blood chemistry assays. Serum was also snap-frozen in microfuge tubes in liquid nitrogen and stored at −70°C until being assayed. All samples were subjected to a single freeze-thaw cycle. Mouse clinical blood chemistries were analyzed using a Beckman Coulter DXC 600. Data was tested for significance using a one-way ANOVA with Tukey's posthoc comparison. The analysis included 11 wild-type mice, pooled as littermates from each mutant genotype, five *Gars^C201R/+^*, eight *Gars^Nmf249/+^*, and six *Agrn^nmf380/nmf380^* mice. *Agrn* mutant mice ranged in age from postnatal day 17 to 44. These mice were compared to age matched littermate control animals, and independently to the wild-type control animals from the *Gars^Nmf249^* and *Gars^C201R^* litters with similar results. The data from the *Gars* control animals is plotted.

Serum samples from the human subjects were obtained and processed according to the standard clinical laboratory procedures.

### Ascorbate assay

Serum ascorbic acid levels were assayed using a colormetric detection system according to the manufacturers instructions (Abcam, 65656). A 96-well plate format was used and results were read on a photospectrometer plate reader. Fifty microliters of serum was used in each assay to be well within the sensitivity of the assay and the linear range of the standard curve. Assay attempts on tissue such as kidney, liver, and spinal cord, failed due to precipitate formation that prevented accurate photospectroscopy readings. Data was tested for significance using a Student's *t*-test. Analysis was performed on four *Gars^Nmf249/+^*, three *Gars^C201R/+^*, and six wild-type mice pooled as littermates of the mutants.

### Metabolomic analysis

#### Metabolite analysis

Metabolomic profiling analysis was performed by Metabolon as previously described (Reitman et al., 2011). The methods below describing sample accessioning, sample preparation, ultrahigh performance liquid chromatography/mass spectrometry, gas chromatography/mass spectrometry, quality assurance/quality control, and data extraction and compound identification were provided by Metabolon and describe their methods, work flow, and analysis. The samples used in this study were analyzed using these standardized methods.

#### Sample accessioning

Each sample received was accessioned into the Metabolon LIMS system and was assigned by the LIMS a unique identifier that was associated with the original source identifier only. This identifier was used to track all sample handling, tasks, results etc. The samples (and all derived aliquots) were tracked by the LIMS system. All portions of any sample were automatically assigned their own unique identifiers by the LIMS when a new task is created; the relationship of these samples is also tracked. All samples were maintained at −80°C until processed.

#### Sample preparation

Samples were prepared using the automated MicroLab STAR^®^ system from Hamilton Company. A recovery standard was added prior to the first step in the extraction process for quality control (QC) purposes. Sample preparation was conducted using aqueous methanol extraction process to remove the protein fraction while allowing maximum recovery of small molecules. The resulting extract was divided into four fractions: one for analysis by UPLC/MS/MS (positive mode), one for UPLC/MS/MS (negative mode), one for GC/MS, and one for backup. Samples were placed briefly on a TurboVap^®^ (Zymark) to remove the organic solvent. Each sample was then frozen and dried under vacuum. Samples were then prepared for the appropriate instrument, either UPLC/MS/MS or GC/MS.

#### Ultrahigh performance liquid chromatography/mass spectroscopy (UPLC/MS/MS)

The LC/MS portion of the platform was based on a Waters ACQUITY ultra-performance liquid chromatography (UPLC) and a Thermo-Finnigan linear trap quadrupole (LTQ) mass spectrometer, which consisted of an electrospray ionization (ESI) source and linear ion-trap (LIT) mass analyzer. The sample extract was dried then reconstituted in acidic or basic LC-compatible solvents, each of which contained eight or more injection standards at fixed concentrations to ensure injection and chromatographic consistency. One aliquot was analyzed using acidic positive ion optimized conditions and the other using basic negative ion optimized conditions in two independent injections using separate dedicated columns. Extracts reconstituted in acidic conditions were gradient eluted using water and methanol containing 0.1% formic acid, while the basic extracts, which also used water/methanol, contained 6.5 mM ammonium bicarbonate. The MS analysis alternated between MS and data-dependent MS2 scans using dynamic exclusion (Evans et al., 2009). Raw data files are archived and extracted as described below.

#### Gas chromatography/mass spectroscopy (GC/MS)

The samples destined for GC/MS analysis were re-dried under vacuum desiccation for a minimum of 24 h prior to being derivatized under dried nitrogen using bistrimethyl-silyl-triflouroacetamide (BSTFA). The GC column was 5% phenyl and the temperature ramp was from 40° to 300°C in a 16-min period. Samples were analyzed on a Thermo-Finnigan Trace DSQ fast-scanning single-quadrupole mass spectrometer using electron impact ionization. The instrument was tuned and calibrated for mass resolution and mass accuracy on a daily basis. The information output from the raw data files was automatically extracted as discussed below.

#### Quality assurance/quality control

For QA/QC purposes, additional samples were included with each day's analysis. These samples included extracts of a pool of well-characterized human plasma, extracts of a pool created from a small aliquot of the experimental samples, and process blanks. QC samples were spaced evenly among the injections and all experimental samples were randomly distributed throughout the run. A selection of QC compounds was added to every sample for chromatographic alignment, including those under test. These compounds were carefully chosen so as not to interfere with the measurement of the endogenous compounds.

#### Data extraction and compound identification

Raw data was extracted, peak-identified and QC processed using Metabolon's hardware and software. These systems are built on a web-service platform utilizing Microsoft's .NET technologies, which run on high-performance application servers and fiber-channel storage arrays in clusters to provide active failover and load-balancing (Dehaven et al., 2010). Compounds were identified by comparison to library entries of purified standards or recurrent unknown entities. Metabolon maintains a library based on authenticated standards that contains the retention time/index (RI), mass to charge ratio (m/z), and chromatographic data (including MS/MS spectral data) on all molecules present in the library. Furthermore, biochemical identifications are based on three criteria: retention index within a narrow RI window of the proposed identification, nominal mass match to the library ±0.2 amu, and the MS/MS forward and reverse scores between the experimental data and authentic standards. The MS/MS scores are based on a comparison of the ions present in the experimental spectrum to the ions present in the library spectrum. While there may be similarities between these molecules based on one of these factors, the use of all three data points can be utilized to distinguish and differentiate biochemicals. More than 2400 commercially available purified standard compounds have been acquired and registered into LIMS for distribution to both the LC and GC platforms for determination of their analytical characteristics. Comparisons between groups were made using Welch's two-sample *t*-test and both *P*-values (significance) and q values (false discovery rate) are reported. Changes with a *P*<0.05 and q<0.10 were considered significant. Significant correlations between groups (R-squared >0.5) were observed only with genotype (*Gars^Nmf249/+^* vs *Gars^+/+^*), and not with sex, date of birth, or litter.

### Statistical analysis of metabolomics data

A total of 20 samples (12 control and eight mutant samples) with a total of 289 metabolites were analyzed with the null hypothesis of no difference between the mutant and control samples. Various different exploratory, univariate and multivariate and machine learning methods were used in the data analysis from publically available software packages Metaboanalyst (Xia et al., 2012, 2009).

Chemical structures were known for 228 of the 289 metabolites detected. Seventeen metabolites (16 with known structure, one unknown structure, Table S2) had 50% or more values missing in either mutant or control or both samples and were discarded from further analysis. The final data matrix contained 20 samples and 272 metabolites. Missing values were imputed using the k-nearest neighbors (KNN) method (Stacklies et al., 2007). Fold-change analysis was performed to compare absolute group means between the two groups and was thus performed before any normalization step. The data was then log2 transformed and autoscaled (Dieterle et al., 2006; van den Berg et al., 2006). Student's *t*-test, hierarchical clustering analysis using Euclidian distance and Ward methods, heatmap, and principal component analysis (PCA) were performed as exploratory data analysis methods. A machine learning model was developed using support vector machine (SVM) algorithm with linear kernel method to classify the mutant and control samples and rank the importance of metabolites in discriminating the two types of samples. R package classification and regression training ‘caret’ version 5.17-7 was used in SVM model generation (Kuhn, 2008). Receiver operating characteristic curve (ROC) analysis was performed using the R package ROCR version 1.0-5 (Sing et al., 2005) to evaluate the sensitivity/specificity of the classification of the samples as mutant or control based on the metabolite signatures.

A nested cross validation (CV) approach was used in which 50 sets were created by randomly dividing the 20 samples into two parts, training and test. In each of these 50 sets, the SVM model was trained on 70% of the samples, and was tested on the remaining 30% of the samples. The results from the 50 test samples were used to calculate the model performance and create an importance score index to rank the contribution of each of the 272 metabolites in classification of mutant from control samples. Finally, the same analysis of 50 resamples was performed on the dataset with randomly shuffled class labels to compare and contrast the performance of SVM model on a true dataset with a random dataset with no biological signal.
